# The genus *Scaptodrosophila* Duda part II: the *coracina* species group from East Asia, with morphological and molecular evidence (Diptera, Drosophilidae)

**DOI:** 10.3897/zookeys.736.13682

**Published:** 2018-02-08

**Authors:** Yi-Qin Liu, Hong-Wei Chen

**Affiliations:** 1 Department of Entomology, South China Agricultural University, Tianhe, Guangzhou, 510642, China

**Keywords:** China, DNA barcoding, integrated taxonomy, *Scaptodrosophila coracina* species group

## Abstract

Eight new species of the *Scaptodrosophila coracina* species group are described from China: *S. fuscilimba* **sp. n.**, *S. fusciventricula* **sp. n.**, *S. helvpecta* **sp. n.**, *S. longispinata* **sp. n.**, *S. nigrolimbata* **sp. n.**, *S. trivittata* **sp. n.**, *S. ventriobscurata* **sp. n.**, and *S. zebrina* **sp. n.** One known species S. c*oracina* (Kikkawa & Peng) is redescribed. A key to all the examined species in the *coracina* group is provided. Species delimitations have been improved by integrating the DNA sequences with morphological information. The intra- and interspecific pairwise p-distances (proportional distance) are summarized. Some nucleotide sites with fixed status in the alignment of the COI sequences (662 nucleotide sites in length) are used as “pure” molecular diagnostic characters to delineate species in the *coracina* group.

## Introduction

The *Scaptodrosophila coracina* species group includes 14 known species (Bächli 2017). Within this group, *S. coracina* (Kikkawa & Peng, 1938) was found from the Oriental region, while the other species were recorded from the Australasian region: *S. cancellata* (Mather, 1955); *S. claytoni* van Klinken, 1997; *S. ellenae* (Bock, 1980); *S. enigma* (Malloch, 1927); *S. evanescens* van Klinken, 1997; *S. garumga* van Klinken, 1997; *S. howensis* (Parsons & Bock, 1979); *S. nitidithorax* (Malloch, 1927); *S. novamaculosa* (Mather, 1956); *S. precaria* van Klinken, 1997; *S. specensis* (Bock, 1976); *S. subnitida* (Malloch, 1927); *S. lativittata* (Malloch, 1923). The diagnosis of the *coracina* group was revised by Bock (1982) and van Klinken (1997) as following: body length up to ca. 3.5 mm; arista with three or four dorsal and two ventral branches in addition to terminal bifurcation; carina smooth; prescutellar setae usually large; hypandrium with a pair of very large paramedian setae.

In the present study, eight new species discovered from China are described, and one known species is redescribed. DNA barcoding was conducted to evaluate morphological delimitation for the *coracina* group, and for this, a total of 35 COI (mitochondrial cytochrome *c* oxidase subunit I) gene sequences of the above-mentioned nine species are determined (Table 1).

**Table 1.** Specimens of the *coracina* group used for DNA barcoding.

Species	Specimen number	Sex	BOLD Process ID	GenBank accession number	Collection site
*S. coracina* –1	111019	♂	BDORM062-17	MF069139	Iriomote Is., Okinawa, Japan
*S. coracina* –2	111023	♀	BDORM063-17	MF069140	Iriomote Is., Okinawa, Japan
*S. coracina* –3	111026	♂	BDORM064-17	MF069141	Samage, Weixi, Yunnan, China
*S. coracina* –4	111029	♀	BDORM065-17	MF069142	Samage, Weixi, Yunnan, China
*S. coracina* –5	111031	♂	BDORM066-17	MF069143	Mayanghe, Yanhe, Guizhou, China
*S. coracina* –6	111032	♂	BDORM067-17	MF069144	Mayanghe, Yanhe, Guizhou, China
*S. coracina* –7	111042	♀	BDORM068-17	MF069145	Mayanghe, Yanhe, Guizhou, China
*S. fuscilimba* sp. n. –1	110975	♂	BDORM039-17	MF066953	Mengdong, Cangyuan, Yunnan, China
*S. fuscilimba* sp. n. –2	110976	♂	BDORM040-17	MF066954	Mengdong, Cangyuan, Yunnan, China
*S. fuscilimba* sp. n. –3	110979	♀	BDORM041-17	MF066955	Mengdong, Cangyuan, Yunnan, China
*S. fusciventricula* sp. n. –1	110987	♂	BDORM044-17	MF066956	Menglun, Mengla, Yunnan, China
*S. fusciventricula* sp. n. –2	110988	♂	BDORM045-17	MF066957	Menglun, Mengla, Yunnan, China
*S. helvpecta* sp. n. –1	110957	♂	BDORM033-17	MF066958	Wuzhishan, Hainan, China
*S. helvpecta* sp. n. –2	110958	♀	BDORM034-17	MF066959	Wuzhishan, Hainan, China
*S. helvpecta* sp. n. –3	110961	♂	BDORM035-17	MF066960	Menglun, Mengla, Yunnan, China
*S. helvpecta* sp. n. –4	110973	♂	BDORM038-17	MF066961	Likan, Ximeng, Yunnan, China
*S. helvpecta* sp. n. –5	110965	♂	BDORM036-17	MF066962	Guanlei, Mengla, Yunnan, China
*S. helvpecta* sp. n. –6	110970	♀	BDORM037-17	MF066963	Guanlei, Mengla, Yunnan, China
*S. longispinata* sp. n. –1	110994	♂	BDORM050-17	MF066964	Menglun, Mengla, Yunnan, China
*S. longispinata* sp. n. –2	110991	♂	BDORM048-17	MF066965	Menglun, Mengla, Yunnan, China
*S. longispinata* sp. n. –3	110992	♂	BDORM049-17	MF066966	Menglun, Mengla, Yunnan, China
*S. nigrolimbata* sp. n. –1	110989	♂	BDORM046-17	MF066967	Likan, Ximeng, Yunnan, China
*S. nigrolimbata* sp. n. –2	110990	♂	BDORM047-17	MF066968	Likan, Ximeng, Yunnan, China
*S. trivittata* sp. n. –1	110985	♂	BDORM043-17	MF066969	Mengdong, Cangyuan, Yunnan, China
*S. trivittata* sp. n. –2	110984	♂	BDORM042-17	MF066970	Mengdong, Cangyuan, Yunnan, China
*S. ventriobscurata* sp. n. –1	110995	♂	BDORM051-17	MF066971	Xincheng, Yingjiang, Yunnan, China
*S. ventriobscurata* sp. n. –2	111005	♂	BDORM055-17	MF066972	Arboretum, Ruili, Yunnan, China
*S. ventriobscurata* sp. n. –3	111006	♂	BDORM056-17	MF066973	Husa, Longchuang, Yunnan, China
*S. ventriobscurata* sp. n. –4	110998	♀	BDORM052-17	MF066974	Xincheng, Yingjiang, Yunnan, China
*S. ventriobscurata* sp. n. –5	111002	♂	BDORM053-17	MF066975	Wangtianshu, Mengla, Yunnan, China
*S. zebrina* sp. n. –1	111007	♂	BDORM057-17	MF066976	Menglun, Mengla, Yunnan, China
*S. zebrina* sp. n. –2	111008	♂	BDORM058-17	MF066977	Menglun, Mengla, Yunnan, China
*S. zebrina* sp. n. –3	111011	♀	BDORM059-17	MF066978	Menglun, Mengla, Yunnan, China
*S. zebrina* sp. n. –4	111017	♀	BDORM061-17	MF066979	Wangtianshu, Mengla, Yunnan, China
*S. zebrina* sp. n. –5	111015	♂	BDORM060-17	MF066980	Wangtianshu, Mengla, Yunnan, China

## Materials and methods

### Specimens

The *coracina* group flies were collected by net sweeping from tussocks and tree trunks near streams in forests. All the examined specimens were preserved in 75% ethanol.

### Species identification

The specimens were first identified as of the *coracina* group in light of morphology referring to Bock’s (1982) and van Klinken’s (1997) diagnoses. Then, they were examined for morphometric characters and detailed structures of terminalia, and sorted into putative species. For each of these putative species, representative specimens suitable for DNA sequencing were selected, considering also the numbers, geographical origins, and genders of available specimens. The methods of Liu et al. (2017) on morphology and molecular phylogenetics were followed.

All the sequences determined in this study were submitted to BOLD (The Barcode of Life Data system) and GenBank (Table 1). A total of 35 COI sequences of the *coracina* group were examined and aligned in MEGA 7.0 (Kumar et al. 2016). Then, calculation of the inter- and intraspecific genetic distances, construction of the neighbor-joining (NJ) tree and the character-based species delimitation were performed in MEGA 7.0. In this paper, four species described in Liu et al. (2017), *S. maculata* (GenBank accession number for COI sequence: KR070820), *S. melanogaster* (KR070823), *S. nigricostata* (KR070829), and *S. obscurata* (KR070838) were used as outgroups.

### Description of species

An Mshot Camera was used to microphotograph specimens, photograph illustration and line drawings were processed with Adobe Photoshop 7.0 and Easy PaintTool SAI Ver.1.0.0. Zhang and Toda (1992) and Chen and Toda (2001) are followed for the definitions of measurements, indices and abbreviations.

The type specimens were deposited in Department of Entomology, South China Agricultural University, Guangzhou, China (**SCAU**).

## Results

The alignment of the 35 COI sequences spanned 662 nucleotide sites in length, with 195 variable sites, among which 188 were parsimony informative. The inter- and intraspecific p-distances in the *coracina* group are shown in Table 2. In most cases, the intraspecific p-distances in the *coracina* group were range from 0 to 2.9%, while the largest intraspecific p-distance in the *coracina* group was found in S. cor*acina* (= 5.6 %). The smallest interspecific p-distance was found between *S. fuscilimba* sp. n. and *S. helvpecta* sp. n. (= 4.7 %).

**Table 2.** Summary of intra- and interspecific genetic distances in the *S. coracina* group.

Species	N	Intraspecific genetic distances	Interspecific genetic distances
		Min. / Max. / Mean ± SD	Min. / Max. / Mean ± SD
*S. coracina*	7	0.006/0.056/0.0370 ± 0.017	0.094/0.137/0.120 0.012
*S. fuscilimba* sp. n.	3	0.002/ 0.003/ 0.002 ± 0.001	0.047/ 0.139/ 0.110 ± 0.032
*S. fusciventricula* sp. n.	2	0.000/ 0.000/ NA	0.091/ 0.133/ 0.113 ± 0.014
*S. helvpecta* sp. n.	6	0.000/ 0.011/ 0.005 ± 0.004	0.047/ 0.133/ 0.107 ± 0.026
*S. longispinata* sp. n.	3	0.002/ 0.006/ 0.004 ± 0.002	0.080/ 0.127/ 0.108 ± 0.015
*S. nigrolimbata* sp. n.	2	0.000/ 0.000/ 0.000	0.121/ 0.144/ 0.133 ± 0.006
*S. trivittata* sp. n.	2	0.005/ 0.005/ NA	0.065/ 0.139/ 0.107 ± 0.025
*S. ventriobscurata* sp. n.	5	0.002/ 0.008/ 0.005 ± 0.002	0.008/ 0.137/ 0.107 ± 0.013
*S. zebrina* sp. n.	5	0.002/ 0.029/ 0.013 ± 0.013	0.100/ 0.144/ 0.121 ± 0.011

N – the numbers of COI sequences involved in distance calculation; Min. – minimum; Max. – maximum; SD – standard deviation; NA – not applicable.

**Figure 1.**
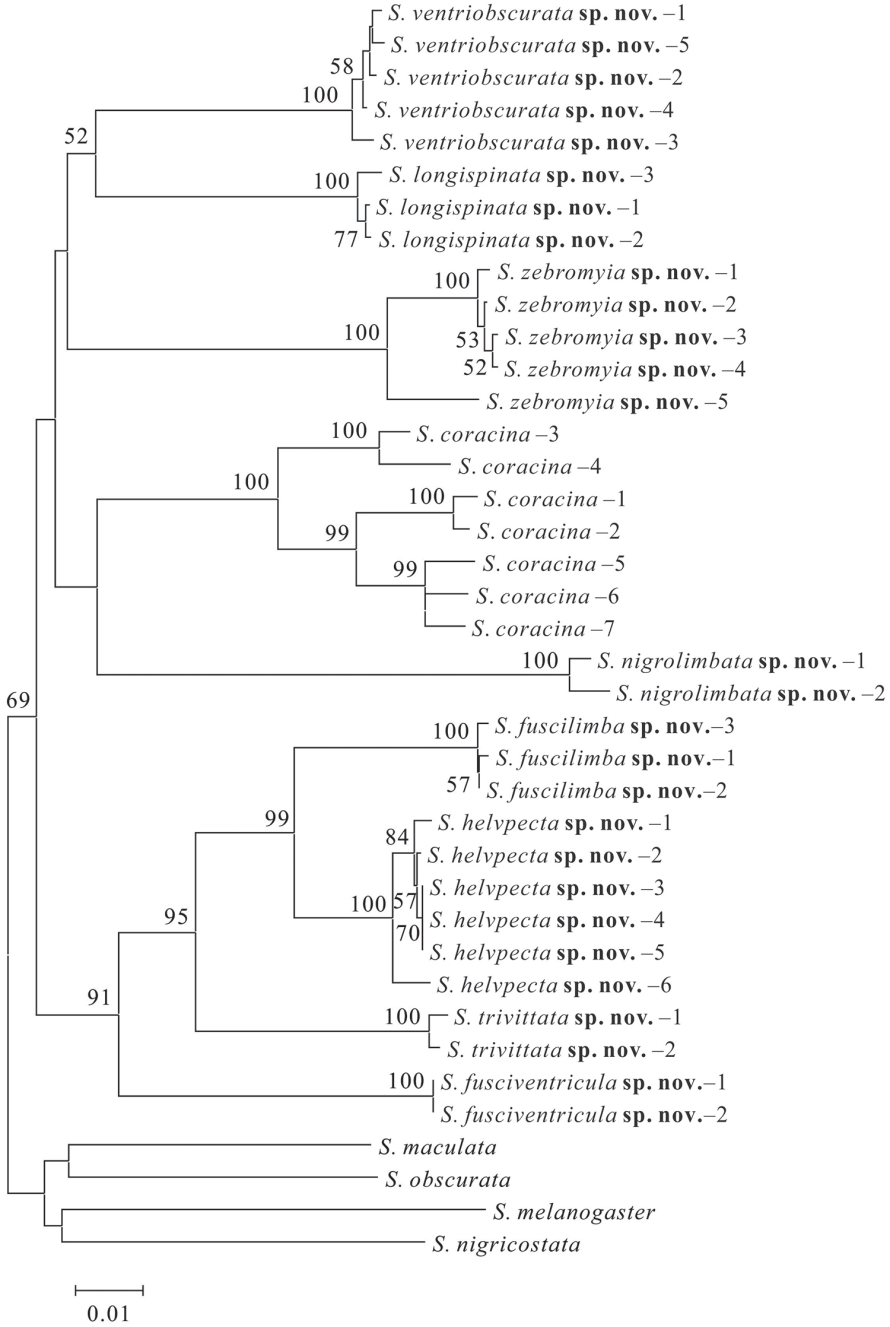
Neighbor-joining (NJ) tree of the *coracina* group. The numbers around the nodes are bootstrap percentages (BP). BP values lower than 50 are not shown.

**Figure 2.**
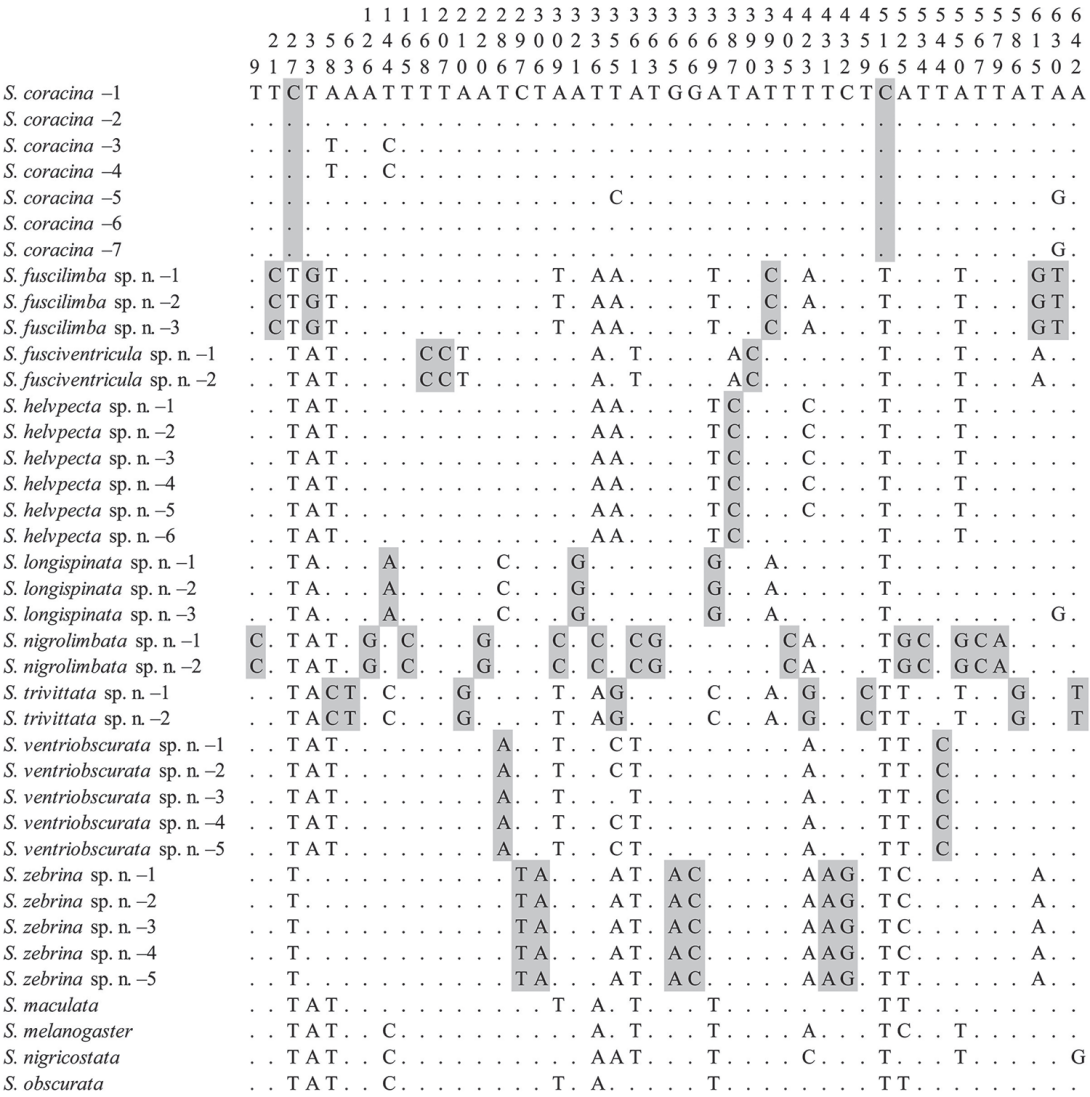
Diagnostic nucleotide sites in the alignment of COI sequences (662 bp in length) of the *coracina* group. Numbers at the top show the positions of the sites in the alignment. Shaded sites are diagnostic for each species. Hyphens (-) indicate missing data.

The NJ tree was shown in Fig. 1. In this tree, each morphologically recognized species was strongly supported [bootstraps percentage (BP) = 100, and them formed a monophyletic group with respect to the outgroups (BP = 69)]. Fig. 2 shows nucleotides at the sites of “pure” diagnostics for each species of the *coracina* group in this study. At least one diagnostic site was recognized for each species.

## Taxonomy

### Scaptodrosophila coracina species group

Taxon classificationAnimaliaDipteraDrosophilidae

Scaptodrosophila coracina species group Mather 1955: 550; Bock 1982: 70; van Klinken 1997: 424.

#### Diagnosis

(modified from Bock 1982 and van Klinken 1997). Body yellowish brown to black; arista with three or four dorsal and two ventral branches in addition to terminal bifurcation; facial carina narrow and flat; prescutellar setae usually large, as long as anterior dorsocentral setae; hypandrium usually with a pair of very large paramedian setae.

#### Description.

**Figure 3.**
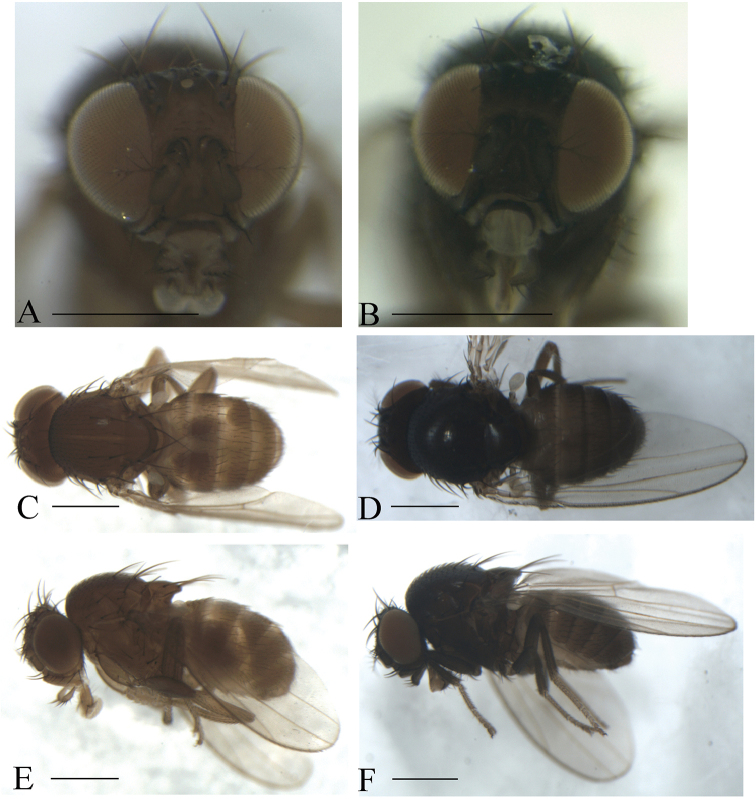
Frontal, dorsal, and lateral views of male. *Scaptodrosophila coracina* from Okinawa, Japan (**A, C, E**) and Guizhou, China (**B, D, F**). Scale bars: 0.5 mm.

**Figure 4.**
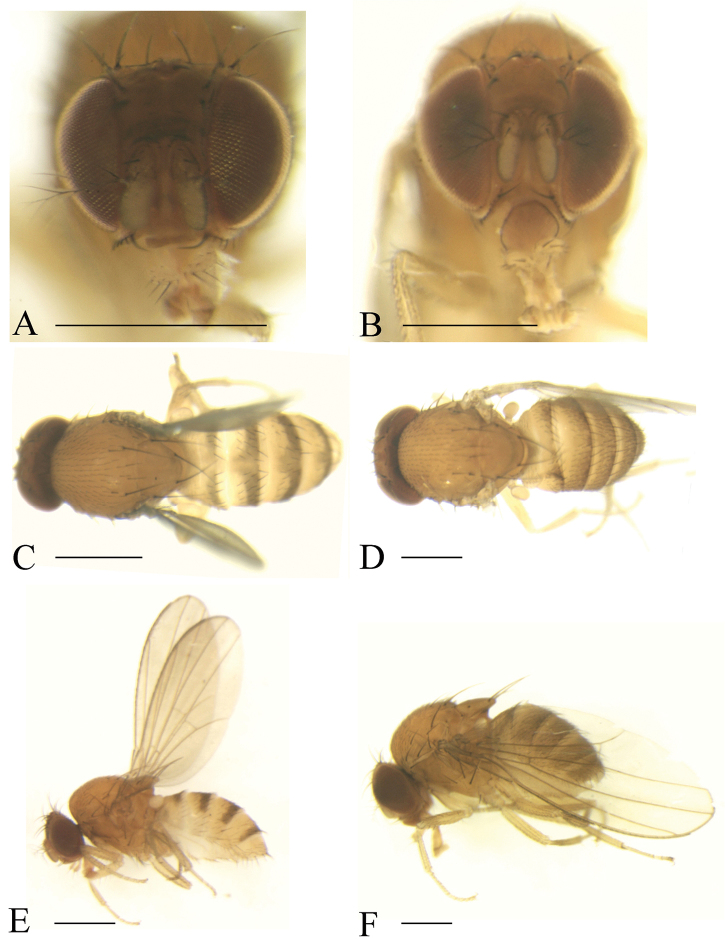
Frontal, dorsal, and lateral views of male. *Scaptodrosophila fuscilimba* sp. n. (**A, C, E**); *S. fusciventricula* sp. n. (**B, D, F**). Scale bars: 0.5 mm.

**Figure 5.**
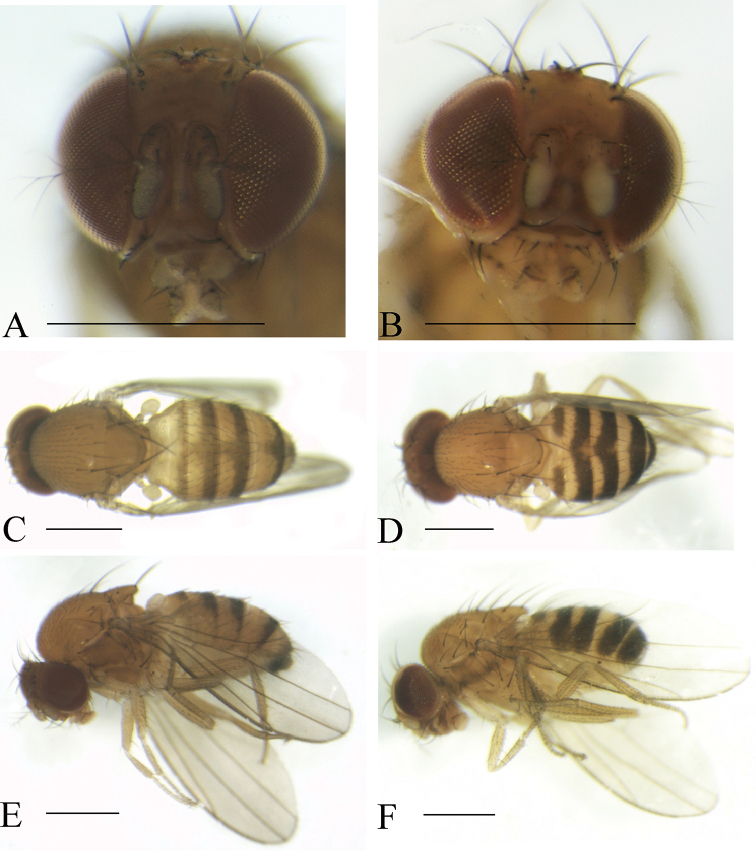
Frontal, dorsal, and lateral views of male. *Scaptodrosophila helvpecta* sp. n. (**A, C, E**); *S. longispinata* sp. n. (**B, D, F**). Scale bars: 0.5 mm.

**Figure 6.**
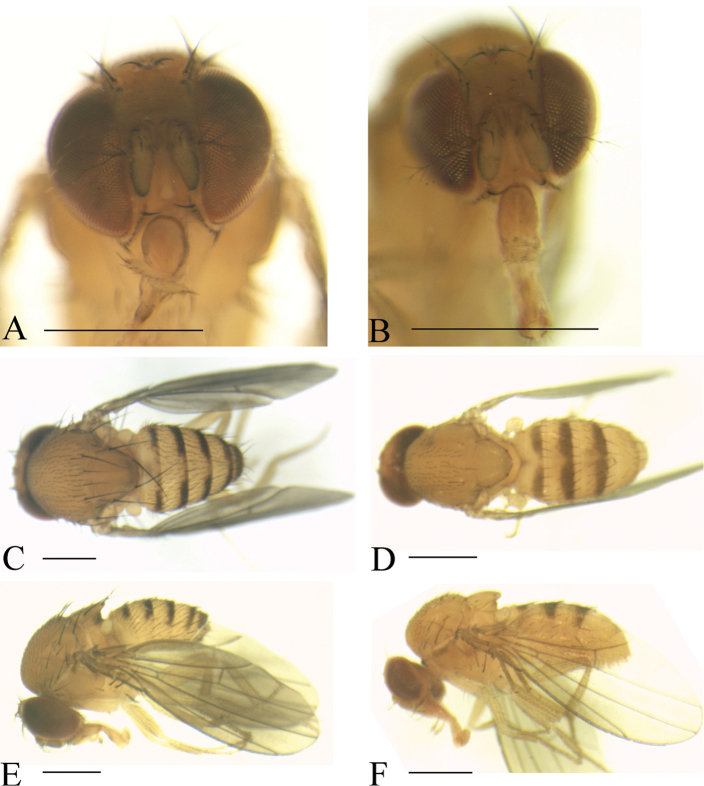
Frontal, dorsal, and lateral views of male. *Scaptodrosophila nigrolimbata* sp. n. (**A, C, E**); *S. trivittata* sp. n. (**B, D, F**). Scale bars: 0.5 mm.

**Figure 7.**
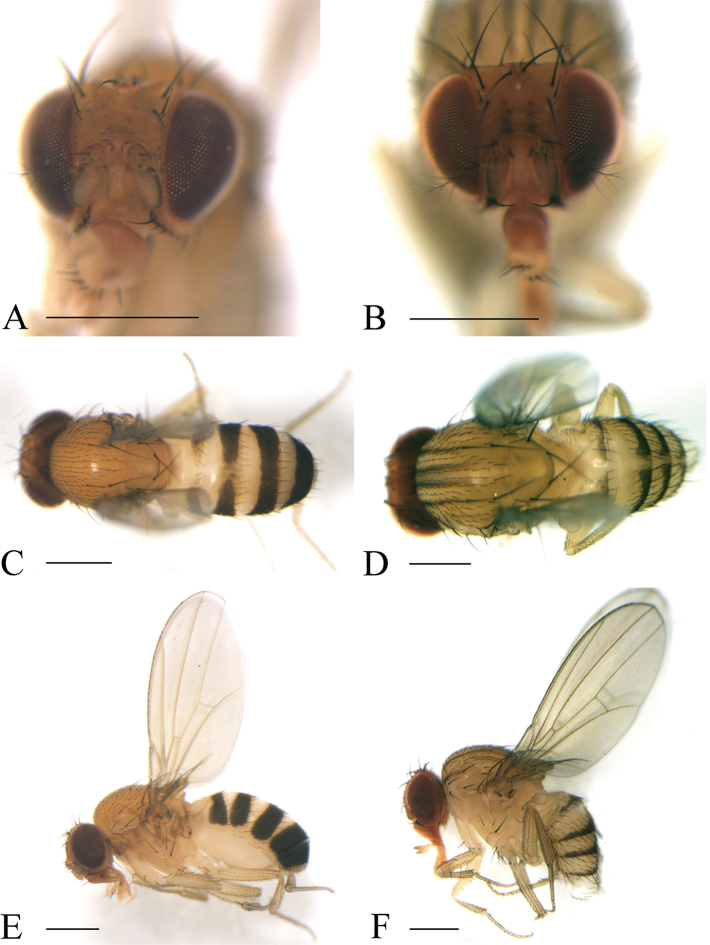
Frontal, dorsal, and lateral views of male. *Scaptodrosophila ventriobscurata* sp. n. (**A, C, E**); *S. zebrina* sp. n. (**B, D, F**). Scale bars: 0.5 mm.

Male and female: ***Head*** (Figs 3–7A, B): Eyes red to brownish red. Ocellar triangle yellowish brown to brown, mostly with three pairs of setae above ocellar setae. Frons narrower than 1/2 width of head, with a few minute setulae medially. Anterior reclinate orbital setae usually outside and close to proclinate orbital setae; posterior reclinate orbital seta larger than others. Face usually yellowish brown to black. Clypeus mostly yellowish brown to brown. Palpus usually yellowish brown. Vibrissa prominent; subvibrissal setae small. Gena and postgena narrow.

***Thorax*** (Figs 3–7C–F): Mesonotum yellowish brown to black, sometimes with longitudinal stripe(s). Postpronotal lobe mostly yellowish to dark brown, with three long setae, and a few of shorter setae. Acrostichal setulae mostly in ca. eight to ten regular rows. Pleura mostly yellowish brown to dark brown. One small proepisternal seta. Katepisternum medially with three large setae and some small ones. Scutellum yellowish brown to black. Wing hyaline, sometimes infuscate. Basal medial-cubital crossvein absent. R_4+5_ nearly parallel with M_1_ distally. Halter mostly white to yellowish. Legs mostly yellowish brown to black.

***Abdomen*** (Figs 3–7C–F): Tergites usually yellowish brown to black, with dark brown caudal bands.

**Figure 8.**
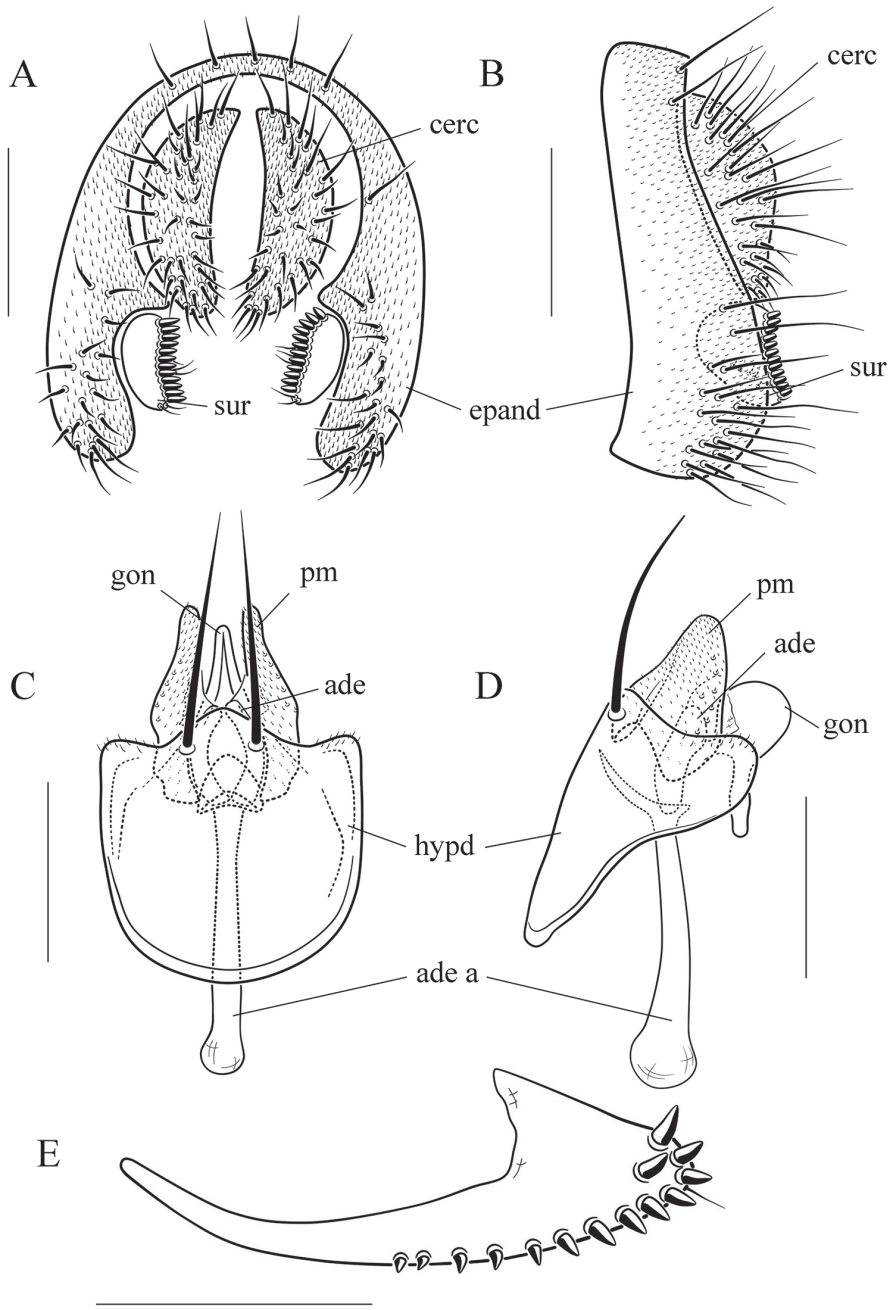
*Scaptodrosophila coracina* (Kikkawa & Peng, 1938). **A** and **B** epandrium (epand), surstylus (sur), and cercus (cerc) (posterior and lateral views, respectively) **C** and **D** hypandrium (hypd), parameres (pm), gonopods (gon), aedeagus (aed), and aedeagal apodeme (aed a) (ventral and lateral views, respectively) **E** oviscapt (lateral view). Scale bars: 0.1 mm.

**Figure 9.**
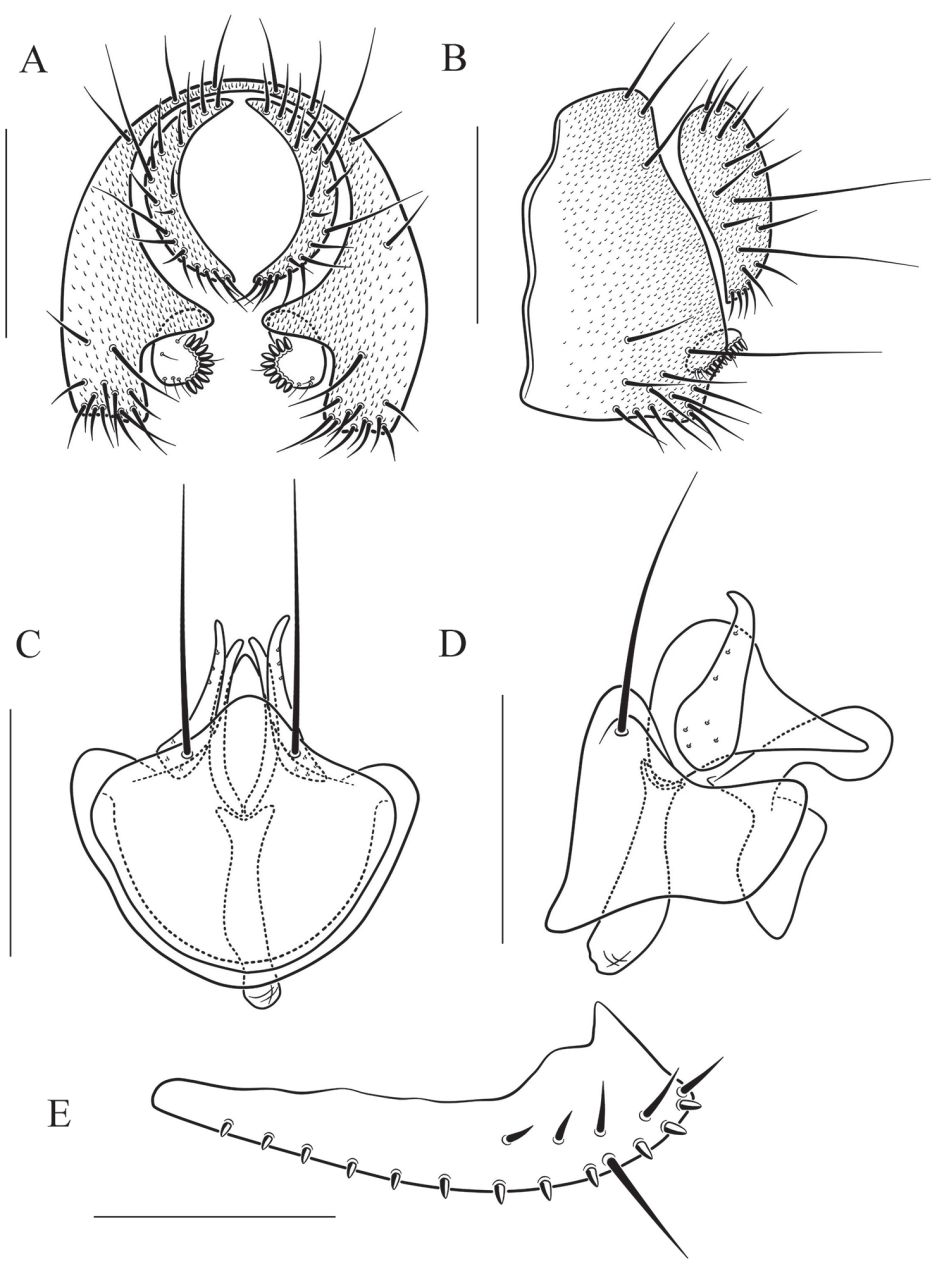
*Scaptodrosophila fuscilimba* sp. n. **A** and **B** epandrium, surstylus, and cercus (posterior and lateral views) **C** and **D** hypandrium, parameres, gonopods, aedeagus, and aedeagal apodeme (ventral and lateral views, respectively) **E** oviscapt (lateral view). Scale bars: 0.1 mm.

**Figure 10.**
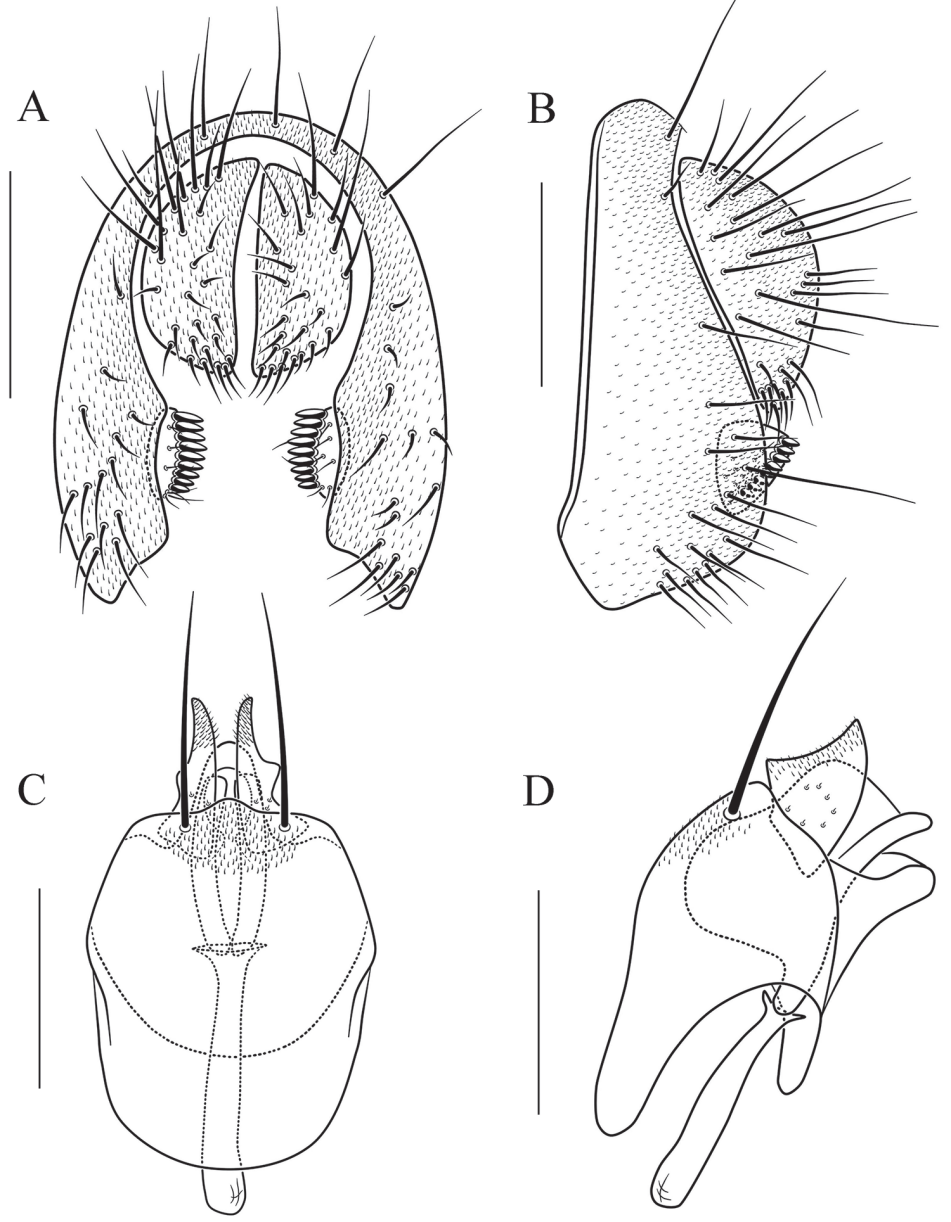
*Scaptodrosophila fusciventricula* sp. n. **A** and **B** epandrium, surstylus, and cercus (posterior and lateral views) **C** and **D** hypandrium, parameres, gonopods, aedeagus, and aedeagal apodeme (ventral and lateral views, respectively). Scale bars: 0.1 mm.

**Figure 11.**
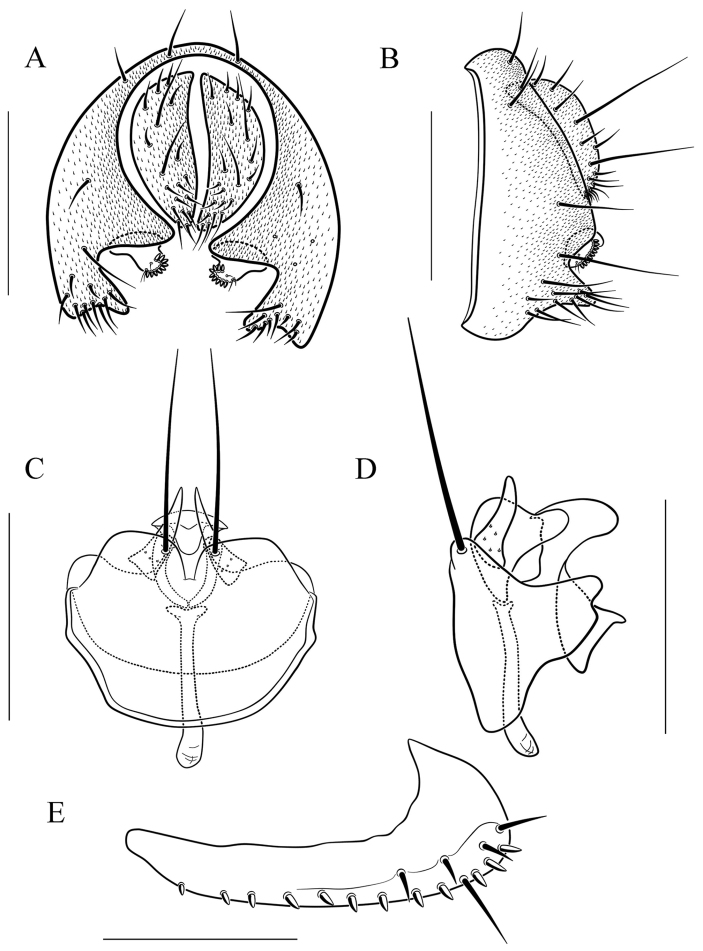
*Scaptodrosophila helvpecta* sp. n. **A** and **B** epandrium, surstylus, and cercus (posterior and lateral views) **C** and **D** hypandrium, parameres, gonopods, aedeagus, and aedeagal apodeme (ventral and lateral views, respectively) **E** oviscapt (lateral view). Scale bars: 0.1 mm.

**Figure 12.**
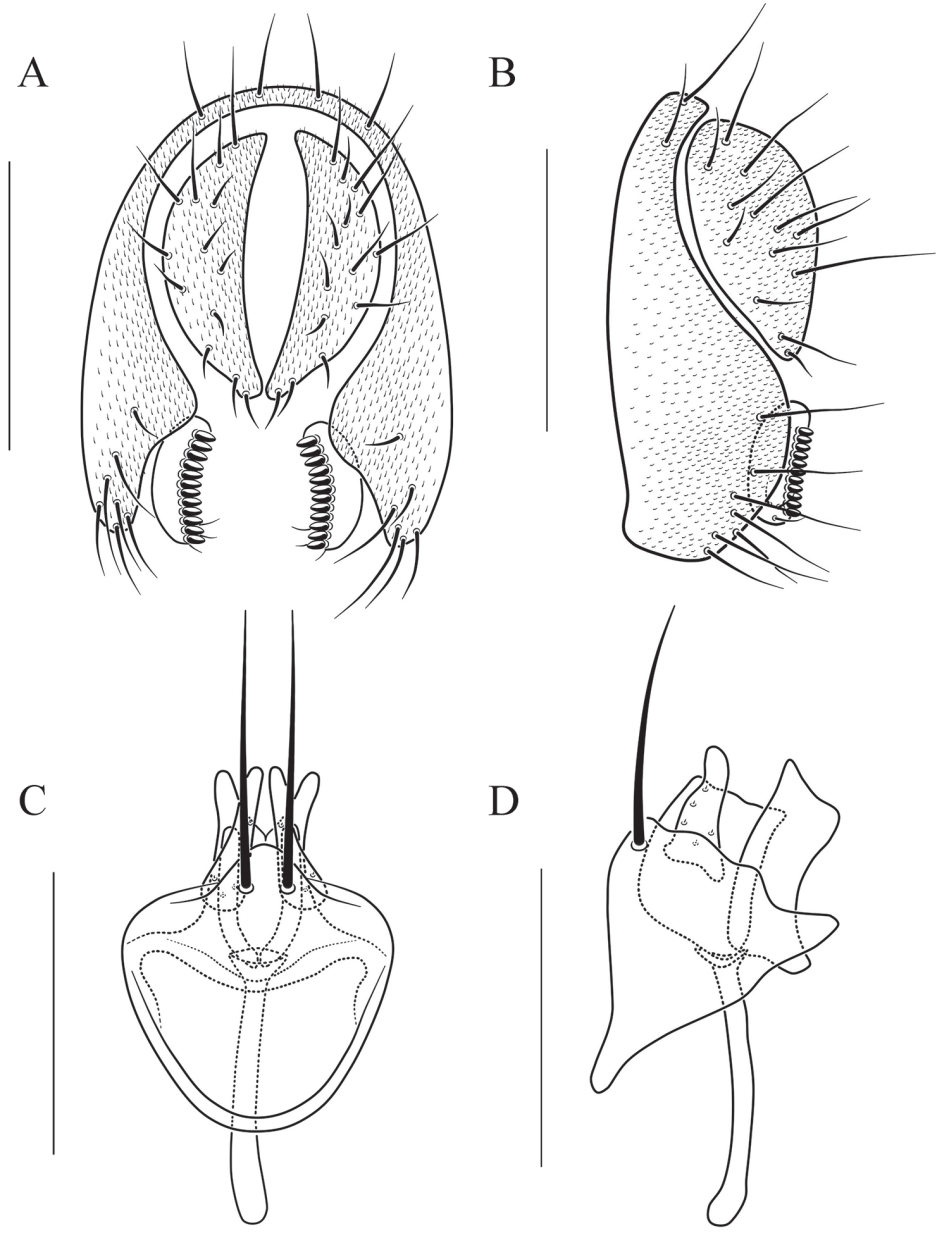
*Scaptodrosophila longispinata* sp. n. **A** and **B** epandrium, surstylus, and cercus (posterior and lateral views) **C** and **D** hypandrium, parameres, gonopods, aedeagus, and aedeagal apodeme (ventral and lateral views, respectively). Scale bars: 0.1 mm.

**Figure 13.**
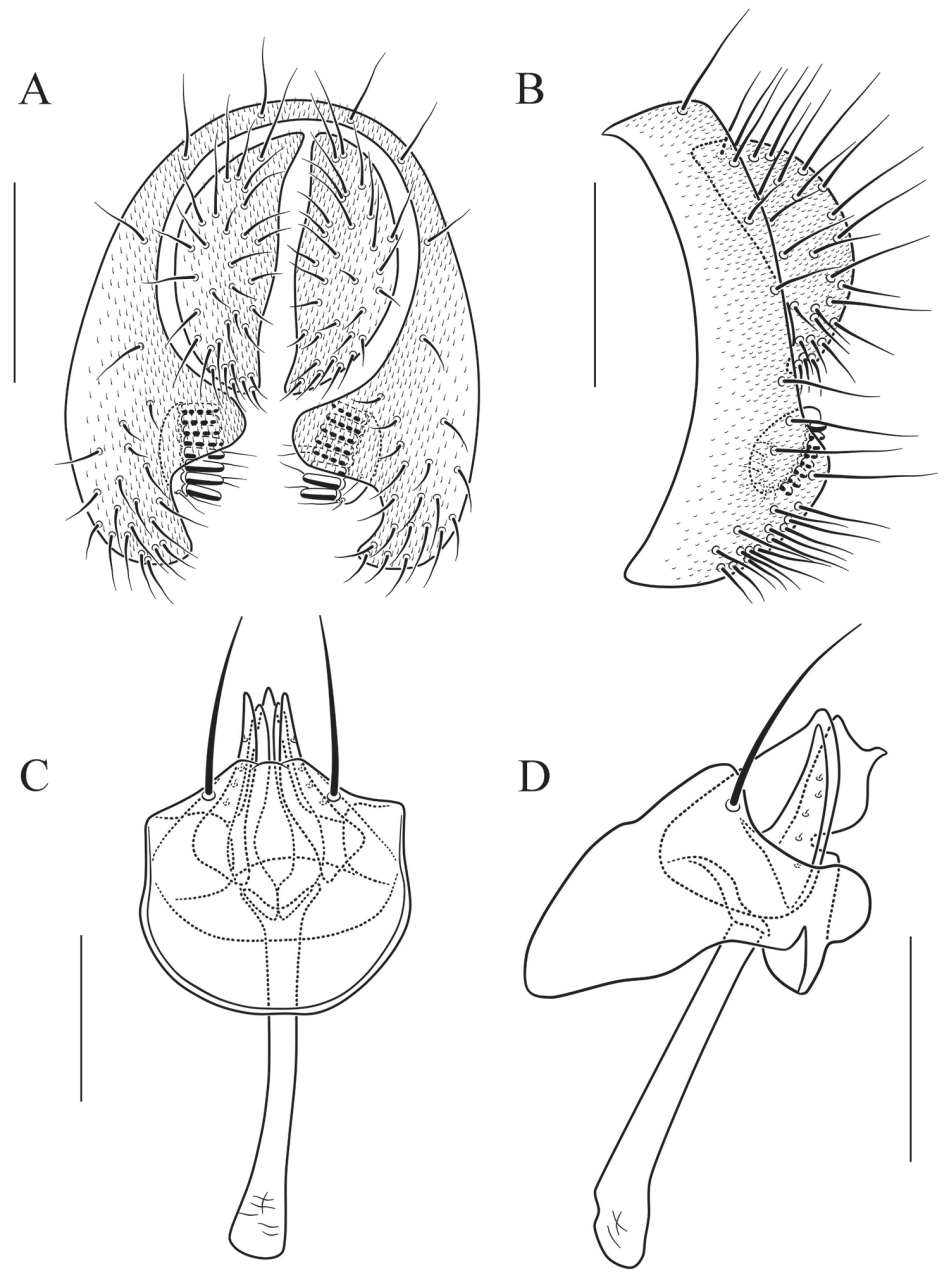
*Scaptodrosophila nigrolimbata* sp. n. **A** and **B** epandrium, surstylus, and cercus (posterior and lateral views) **C** and **D** hypandrium, parameres, gonopods, aedeagus, and aedeagal apodeme (ventral and lateral views, respectively). Scale bars: 0.1 mm.

**Figure 14.**
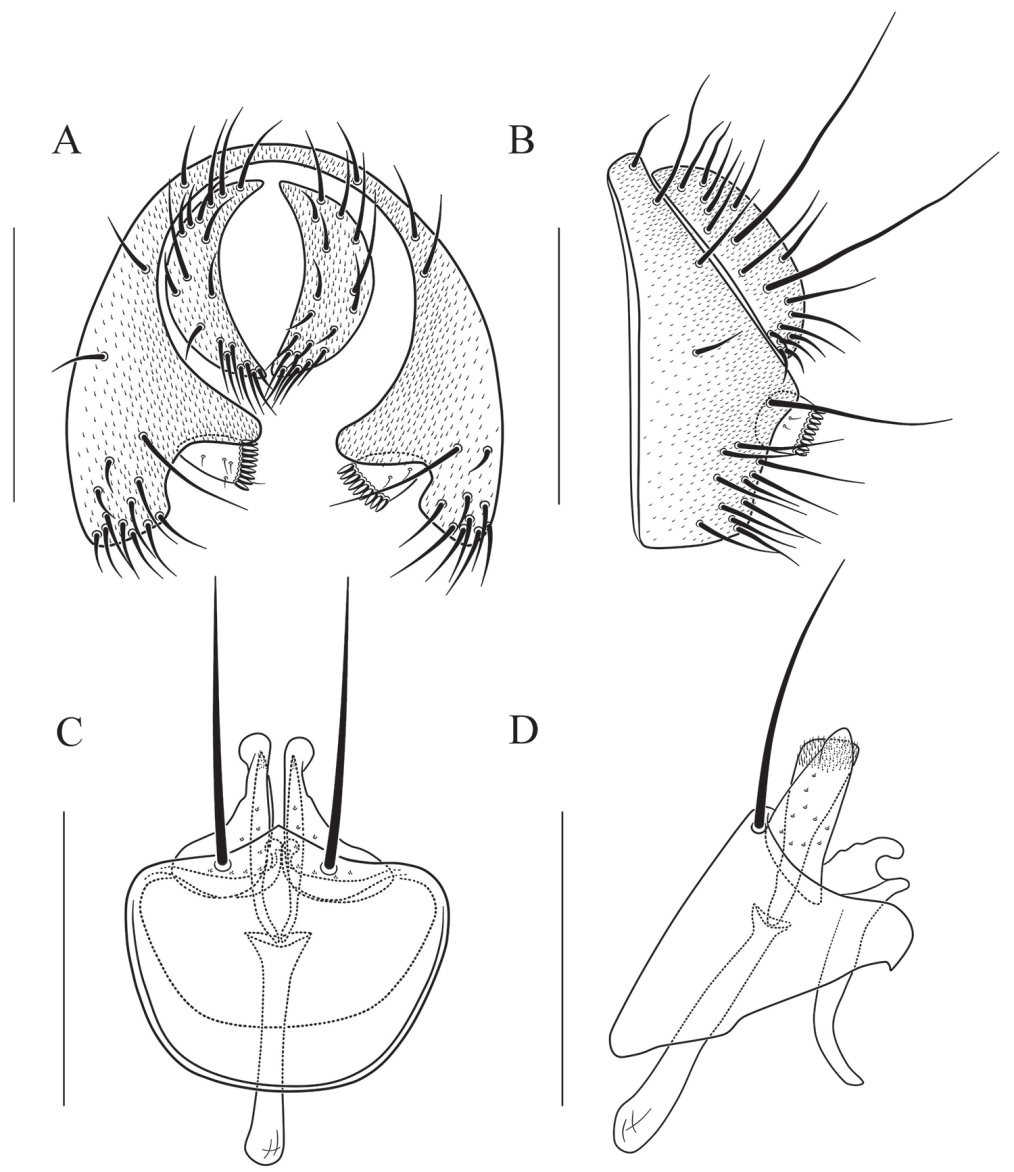
*Scaptodrosophila trivittata* sp. n. **A** and **B** epandrium, surstylus, and cercus (posterior and lateral views) **C** and **D** hypandrium, parameres, gonopods, aedeagus, and aedeagal apodeme (ventral and lateral views, respectively). Scale bars: 0.1 mm.

**Figure 15.**
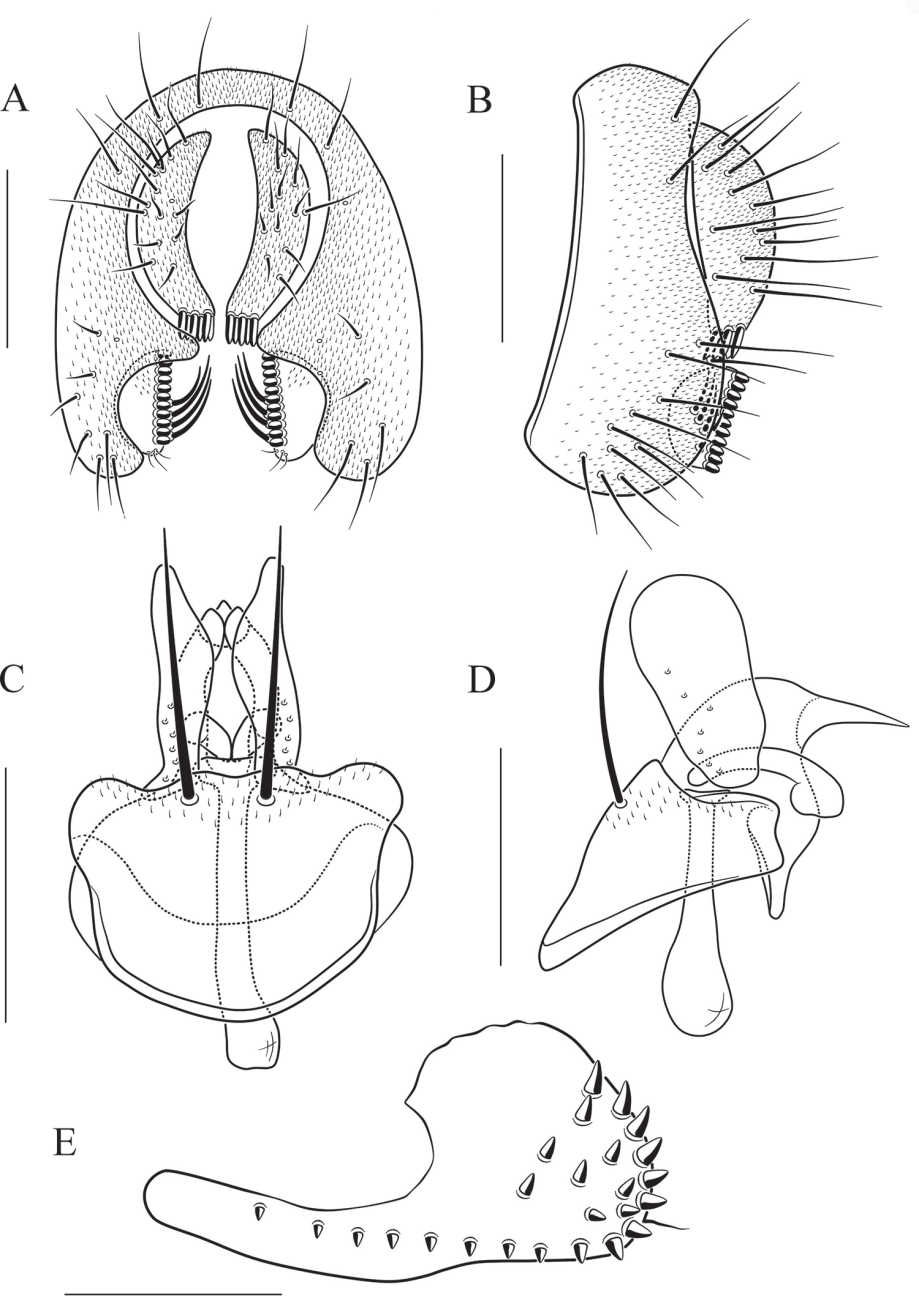
*Scaptodrosophila ventriobscurata* sp. n. **A** and **B** epandrium, surstylus, and cercus (posterior and lateral views) **C** and **D** hypandrium, parameres, gonopods, aedeagus, and aedeagal apodeme (ventral and lateral views, respectively) **E** oviscapt (lateral view). Scale bars: 0.1 mm.

**Figure 16.**
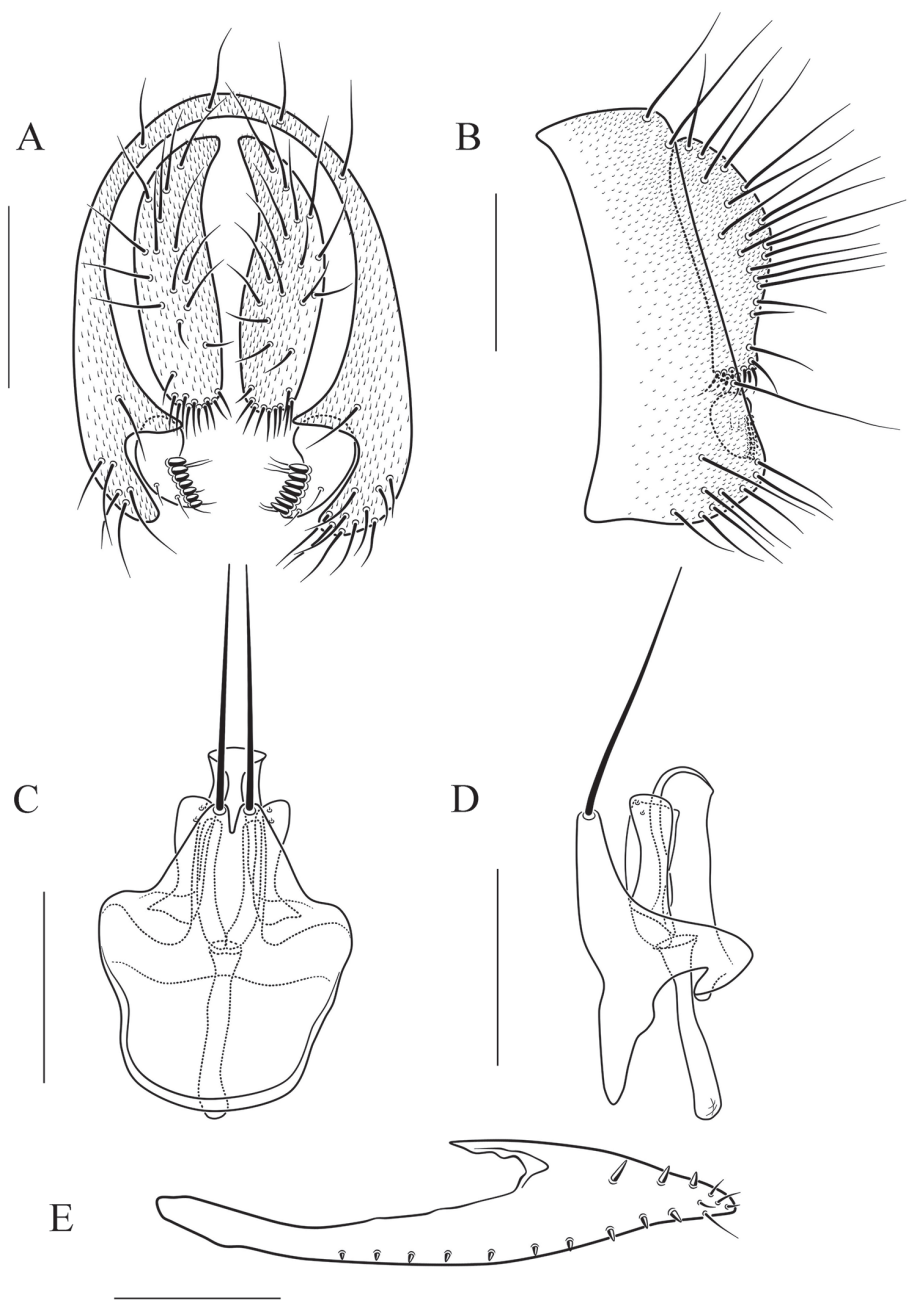
*Scaptodrosophila zebrina* sp. n. **A** and **B** epandrium, surstylus, and cercus (posterior and lateral views) **C** and **D** hypandrium, parameres, gonopods, aedeagus, and aedeagal apodeme (ventral and lateral views, respectively) **E** oviscapt (lateral view). Scale bars: 0.1 mm.

***Male terminalia*** (Figs 8–16A–D): Epandrium usually pubescent, with several setae around anteroventral corner to posterior margin. Surstylus with a row of peg-like prensisetae long caudal margin, several setae on outer and inner surfaces. Cercus separated from epandrium, pubescent, and setigerous. Hypandrium pale brown. Paramere with several sensilla. Gonopods fused with each other, broadened to hood-shaped. Aedeagus bilobed subbasally.

***Female terminalia*** (Figs 8, 9, 11, 15, 16E): Oviscapt mostly yellowish brown to brown, usually broadened subapically in lateral view, and with numerous peg-like ovisensilla.

In the following individual species descriptions, only characters that depart from the above description are provided for brevity.

### Scaptodrosophila coracina

Taxon classificationAnimaliaDipteraDrosophilidae

(Kikkawa & Peng, 1938)

Figs 3 , 8

Drosophila coracina Kikkawa & Peng, 1938: 523.

#### Material examined.

CHINA: 11♂, 5♀ (SCAU, Nos 111031–46), Mayanghe, Yanhe, Guizhou, 28°39'N, 108°16'E, alt. 422–1500 m, 24–25.v.2012, JM Lu; 10♂, 8♀ (SCAU, Nos 111047–64), Jiaoye Park, Kunming, Yunnan, 25°02'N, 102°37'E, alt. 1900 m, 17.vii.2004, JJ Gao; 3♂, 2♀ (SCAU, Nos 111026–30), Samage, Weixi, Yunnan, 27°22'N, 99°51'E, alt. 1900 m, 30.vii.2004, HW Chen. JAPAN: 5♂, 3♀ (SCAU, Nos 111019–25), Iriomote, Okinawa, 24°32'N, 123°88'E, alt. 150 m, 12.v.2001, HW Chen.

#### Diagnosis.

Paramere large, pubescent completely (Fig. 8C, D); aedeagus short, rod-like, apically membranous, fan-like (Fig. 8C, D).

#### Description.

(♂, ♀) ***Head*** (Fig. 3A, B): Frons yellowish brown to dark brown. Pedicel and first flagellomere brown to dark brown. Facial carina brown to dark brown, 1/3 as long as face.

***Thorax*** (Fig. 3C–F): Mesonotum brown or black. Acrostichal setulae in ca. eight to ten irregular rows. Scutellum brown or black. Pleura brownish or dark brown.

***Abdomen*** (Fig. 3C–F): All tergites brown or dark brown.

***Male terminalia*** (Fig. 8A–D): Epandrium with ca. 19 setae along dorsocaudal margin and on ventral portion per side. Surstylus with ca. 12 peg-like prensisetae. Hypandrium pubescent caudolaterally. Paramere with ca. six to eight sensilla. Gonopods apically round in lateral view. Aedeagus lacking pubescence.

***Female terminalia*** (Fig. 8E): Oviscapt triangle-shaped subapically, with one subterminal, trichoid ovisensillum, 14 ovisensilla.

***Measurements*** (range in 4♂, 4♀, in mm): BL = (1.76–2.33, 2.00–2.40), ThL = (0.83–0.93, 0.86–1.00), WL = (1.50–1.83, 1.50–2.00), WW = (0.67–0.87, 0.70–1.00).

***Indices***: arb = 3/2, avd = 0.71–1.00, adf = 1.67–2.33, flw = 1.50–2.00, FW/HW = 0.36–0.43, ch/o = 0.09–0.11, prorb = 0.70–0.88, rcorb = 0.50–0.60, vb = 0.33–0.57, dcl = 0.48–0.69, presctl = 0.31–0.50, sctl = 0.89–1.00, sterno = 0.60–0.83, orbito = 0.60–0.80, dcp = 0.36–0.53, sctlp = 0.83–1.00, C = 1.53–2.00, 4c = 1.38–1.56, 4v = 2.25–2.56, 5x = 1.75–2.33, ac = 2.60–3.00, M = 0.75–0.89, C3F = 0.76–0.83.

#### Distribution.

Widespread in Asia.

### Scaptodrosophila fuscilimba sp. n.

Taxon classificationAnimaliaDipteraDrosophilidae

http://zoobank.org/18DB7FCE-D66E-49DE-8573-E1B08BCFE4A3

Figs 4A, C, E , 9

#### Holotype.

♂ (SCAU, No. 110975): CHINA: Mengdong, Cangyuan, Yunnan, 23°10'N, 99°13'E, alt. 1323 m, 6.v.2016, YL Wang.

#### Paratypes.

CHINA: 3♂, 5♀ (SCAU, Nos 110976–983), same data as holotype, J Huang, YQ Liu, YL Wang, L Zhu.

#### Diagnosis.

Paramere strongly curved in lateral view (Fig. 9D); aedeagus inverted, triangle shaped, tapering dorsally in lateral view (Fig. 9D).

#### Description.

(♂, ♀) ***Head*** (Fig. 4A): Frons brownish. Pedicel brown to dark brown; first flagellomere yellowish, margins black. Facial carina yellowish, long, and narrow, 1/2 as long as face.

***Thorax*** (Fig. 4C, E): Mesonotum yellowish brown. Acrostichal setulae in ca. eight regular rows. Scutellum yellowish brown. Pleura yellowish brown.

***Abdomen*** (Fig. 4C, E): Tergites II to V yellow with dark brown caudal bands; tergites VI yellow.

***Male terminalia*** (Fig. 9A–D): Epandrium with ca. 16 setae near posterior and ventral margins per side. Surstylus with ca. nine peg-like prensisetae. Hypandrium lacking pubescence. Paramere with three medial and four basal sensilla. Gonopods roundly expanded in lateral view. Aedeagus lacking pubescence.

***Female terminalia*** (Fig. 9E): Oviscapt triangle-shaped subapically, with six long subterminal trichoid ovisensilla, and 12 peg-like ovisensilla along ventral margin.

***Measurements*** [holotype ♂ (range in 3♂, 2♀ paratypes), in mm]: BL = 1.87 (1.70–2.13, 1.83–2.03), ThL = 0.80 (0.77–0.90, 0.83–0.90), WL = 1.63 (1.50–1.90, 1.73–1.83), WW = 0.70 (0.67–0.80, 0.70–0.77).

***Indices***: arb = 3/2 (3/2), avd = 0.83 (0.67–0.80), adf = 2.00 (1.67–2.33), flw = 1.67 (1.67–1.83), FW/HW = 0.48 (0.48–0.52), ch/o = 0.13 (0.11–0.13), prorb = 0.86 (0.75–0.93), rcorb = 0.29 (0.29–0.38), vb = 0.40 (0.38–0.46), dcl = 0.38 (0.29–0.42), presctl = 0.25 (0.32–0.43), sctl = 1.07 (0.86–1.17), sterno = 0.57 (0.36–0.45), orbito = 0.38 (0.38–0.50), dcp = 0.30 (0.32–0.40), sctlp = 086 (0.86–1.08), C = 1.87 (1.65–2.00), 4c = 1.36 (1.25–1.42), 4v =2.36 (2.33–2.50), 5x = 2.00 (2.25–2.50), ac = 3.00 (2.73–3.40), M = 0.73 (0.75–0.90), C3F = 0.67 (0.67–0.80).

#### Etymology.

A combination of the Latin words *fuscus* and *limbus*, referring to the brown caudal margins of abdominal tergites.

#### Distribution.

China (Yunnan).

### Scaptodrosophila fusciventricula sp. n.

Taxon classificationAnimaliaDipteraDrosophilidae

http://zoobank.org/97DE8D73-6561-47C5-A35B-B69DB82FFAB1

Figs 4B, D, F , 10

#### Holotype.

♂ (SCAU, No. 110987): CHINA: Menglun, Mengla, Yunnan, 21°55'N, 101°16'E, alt. 680 m, 11.iv.2011, L Wang.

#### Paratype.

CHINA: 1♂ (SCAU, No. 110988), same data as holotype.

#### Diagnosis.

Paramere dolabriform in lateral view, with pubescence apically (Fig. 10C, D); aedeagus very broad and finely curved apically in lateral view (Fig. 10D).

#### Description.

(♂) ***Head*** (Fig. 4B): Frons yellowish brown. Pedicel yellowish brown; first flagellomere yellowish, margins black. Facial carina yellowish, short, narrow, and flat, 1/4 long as face.

***Thorax*** (Fig. 4D, F): Mesonotum yellowish brown. Acrostichal setulae in ca. ten regular rows. Scutellum yellowish brown. Pleura yellowish brown.

***Abdomen*** (Fig. 4 D, F): All tergites brown.

***Male terminalia*** (Fig. 10): Epandrium with ca. 17 setae near posterior and ventral margins per side. Surstylus with ca. nine peg-like prensisetae. Hypandrium with pubescence basomedially. Paramere with seven medial sensilla. Gonopods V-shaped in lateral view. Aedeagus lacking pubescence.

***Measurements*** [holotype ♂ (range in 1♂ paratype), in mm]: BL = 2.33 (2.33), ThL = 1.07 (1.07), WL = 2.43 (2.33), WW = 1.06 (1.00).

***Indices***: arb = 4/2 (3–4/2), avd = 0.89 (0.88), adf = 3.00 (2.67), flw = 2.67 (2.33), FW/HW = 0.46 (0.43), ch/o = 0.08 (0.09), prorb = 0.89 (0.88), rcorb = 0.44 (0.38), vb = 0.29 (0.40), dcl = 0.50 (0.41), presctl = 0.50 (0.41), sctl = 0.85 (1.00), sterno = 0.50 (damaged), orbito = 0.60 (0.40), dcp = 0.38 (0.33), sctlp = 0.90 (1.00), C = 4.17 (3.92), 4c = 0.60 (0.63), 4v = 1.60 (1.63), 5x = 1.14 (1.50), ac = 1.50 (2.00), M = 0.40 (0.47), C3F = 0.33 (0.25).

#### Etymology.

A combination of the Latin words *fuscus* and *ventriculus*, referring to the brown tergites.

#### Distribution.

China (Yunnan).

### Scaptodrosophila helvpecta sp. n.

Taxon classificationAnimaliaDipteraDrosophilidae

http://zoobank.org/C2071DA5-CEBA-4DE6-8D51-FF91AB369366

Figs 5A, C, E , 11

#### Holotype.

♂ (SCAU, No. 110957): CHINA: Wuzhishan, Hainan, 18°48'N, 109°19'E, alt. 440 m, 21.iv.2007, HZ Cao, T Li.

#### Paratypes.

CHINA: 3♀ (SCAU, Nos 110958–60), same data as holotype; 2♂, 2♀ (SCAU, Nos 110961–64), Menglun, Mengla, Yunnan, alt. 570 m, 12.iv.2010, YR Su, L Wang, L Wu; 5♂, 3♀ (SCAU, Nos 110965–72), Guanlei, Mengla, Yunnan, 21°38'N, 101°16'E, alt. 562 m, 20.iv.2016, J Huang, Y Liu, YL Wang, L Zhu; 2♂ (SCAU, Nos 110973, 74), Likan, Ximeng, Yunnan, 22°39'N, 99°36'E, alt. 844 m, 1.v.2016, J Huang, YQ Liu.

#### Diagnosis.

This species is similar to *S. fuscilimba* sp. n. in the pattern on the abdomen tergites and the shape of male terminalia, but can be distinguished from the latter by having the tergite VI yellowish brown with dark brown caudal bands (Fig. 5C, E); paramere small, finger like in lateral view (Fig. 11D); aedeagus inverted triangle shaped and round dorsally in lateral view (Fig. 11D).

#### Description.

(♂, ♀) ***Head*** (Fig. 5A): Frons yellowish brown. Pedicel yellowish brown; first flagellomere grayish, margins black. Facial carina yellowish, long, narrow, and prominent, 1/2 long as face.

***Thorax*** (Fig. 5C, E): Mesonotum yellowish brown. Acrostichal setulae in ca. ten regular rows. Scutellum yellowish brown. Pleura yellowish brown.

***Abdomen*** (Fig. 5C, E): Tergites II to VI yellowish brown with dark brown caudal bands.

***Male terminalia*** (Fig. 11A–D): Epandrium with ca. 14 setae near posterior and ventral margins per side. Surstylus with approximately nine fine peg-like prensisetae. Hypandrium lacking pubescence. Paramere with five sensilla basally. Gonopods elliptically expanded in lateral view. Aedeagus lacking pubescence.

***Female terminalia*** (Fig. 11E): Oviscapt with five long subapical trichoid ovisensilla, and 12 ovisensilla along ventral margin.

***Measurements*** [holotype ♂ (range in 5♂, 5♀ paratypes), in mm]: BL = 1.73 (1.83–2.07, 1.53–2.00), ThL = 0.83 (0.76–0.90, 0.76–0.90), WL = 1.63 (1.53–1.97, 1.43–1.67), WW = 0.73 (0.67–0.77, 0.60–0.70).

***Indices***: arb = 3/2 (3/2), avd = 0.71 (0.50–1.00), adf = 2.33 (1.75–2.67), flw = 2.17 (1.38–2.00), FW/HW = 0.39 (0.41–0.57), ch/o = 0.10 (0.10–0.12), prorb = 0.78 (0.57–1.00), rcorb = 0.22 (0.25–0.38), vb = 0.29 (0.29–0.50), dcl = 0.29 (0.29–0.42), presctl = 0.43 (0.33–0.50), sctl = 1.00 (0.93–1.20), sterno = 0.50 (0.33–0.60), orbito = 0.50 (0.38–0.50), dcp = 0.32 (0.25–0.40), sctlp = 0.76 (0.67–1.14), C = 1.87 (1.63–2.08), 4c = 1.15 (1.18–1.50), 4v =2.08 (2.00–2.45), 5x = 3.33 (2.33–3.33), ac = 3.00 (2.60–3.20), M = 0.77 (0.64–0.90), C3F = 0.60 (0.67–0.77).

#### Etymology.

A combination of the Latin words *helvus* and *pectus*, referring to the yellow thorax.

#### Distribution.

China (Hainan, Yunnan).

### Scaptodrosophila longispinata sp. n.

Taxon classificationAnimaliaDipteraDrosophilidae

http://zoobank.org/5CA6BE46-F90A-4804-9AFB-19ABC3F1DEED

Figs 5B, D, F , 12

#### Holotype.

♂ (SCAU, No. 110991): CHINA: Menglun, Mengla, Yunnan, alt. 570 m, 12.iv.2010, L Wang.

#### Paratypes.

CHINA: 3♂ (SCAU, Nos 110992–994), Menglun, Mengla, Yunnan, alt. 680 m, 11.iv.2010, L Wu.

#### Diagnosis.

Gonopods bilobed apically (Fig. 12D); aedeagus quadrangle in lateral view (Fig. 12D).

#### Description.

(♂) ***Head*** (Fig. 5B): Frons yellowish brown. Pedicel yellow; first flagellomere yellowish white, margins black. Facial carina yellowish, short, and flat, as 1/4 length as face.

***Thorax*** (Fig. 5D, F): Mesonotum yellowish brown. Acrostichal setulae in ca. ten regular rows. Prescutellar setae small. Scutellum yellowish brown. Pleura yellowish brown, with two brown longitudinal stripes.

***Abdomen*** (Fig. 5D, F): Tergites II to V yellow with dark brown caudal bands, the caudal band on tergite II interrupted medially; tergite VI dark brown.

***Male terminalia*** (Fig. 12): Epandrium with nine setae near posterior and ventral margins per side. Surstylus with 12 peg-like prensisetae. Hypandrium lacking pubescence. Paramere with five sensilla medially. Aedeagus lacking pubescence.

***Measurements*** [holotype ♂ (range in 3♂ paratypes), in mm]: BL = 1.77 (1.60–1.90), ThL = 0.83 (0.67–0.73), WL = 1.60 (1.47–1.67), WW = 0.73 (0.70–0.80).

***Indices***: arb = 3/2 (3/2), avd = 0.86 (0.78–0.88), adf = 2.33 (2.33–3.00), flw = 1.67 (1.67), FW/HW = 0.45 (0.43–0.46), ch/o = 0.12 (0.12–0.13), prorb = 0.75 (0.67–0.88), rcorb = 0.25 (0.29–0.38), vb = 0.50 (0.29–0.40), dcl = 0.50 (0.53–0.73), presctl = 0.43 (0.40–0.46), sctl = 1.07 (0.94–1.25), sterno = 0.75 (0.64–0.82), orbito = 0.40 (0.40–0.50), dcp = 0.50 (0.42–0.45), sctlp = 1.00 (0.86–1.00), C = 2.33 (1.93–2.00), 4c = 1.20 (1.33–1.44), 4v = 2.60 (2.60–2.67), 5x = 2.00 (1.75–2.33), ac = 2.40 (2.40–3.25), M = 0.80 (0.78–0.80), C3F = 0.67 (0.58–0.64).

#### Etymology.

A combination of the Latin words *longus* and *spinatus*, referring to the long spines on the hypandrium.

#### Distribution.

China (Yunnan).

### Scaptodrosophila nigrolimbata sp. n.

Taxon classificationAnimaliaDipteraDrosophilidae

http://zoobank.org/C0BEE38A-91B9-4523-8A9D-13FC9484308F

Figs 6A, C, E , 13

#### Holotype.

♂ (SCAU, No. 110989): CHINA: Likan, Ximeng, Yunnan, alt. 844 m, 1.v.2016, J Huang, YQ Liu.

#### Paratype.

CHINA: 1♂ (SCAU, No. 110990), same data as holotype.

#### Diagnosis.

Surstylus with eight very large peg-like prensisetae (Fig. 13A, B); paramere triangle shaped in lateral view, with a small projection subbasally (Fig. 13D); gonopods concave medially in lateral view, with a small projection distally (Fig. 13D); aedeagus triangle shaped in lateral view (Fig. 13D).

#### Description.

(♂) ***Head*** (Fig. 6A): Frons brown. Pedicel yellowish brown; first flagellomere yellowish brown, margins black. Facial carina yellowish, long, narrow, and flat, as 1/2 length as face.

***Thorax*** (Fig. 6C, E): Mesonotum yellowish brown. Acrostichal setulae in ca. eight regular rows. Scutellum yellowish brown. Pleura yellowish brown.

***Abdomen*** (Fig. 6C, E): Tergites II to VI yellow with dark brown caudal bands, the caudal band on tergites II to V interrupted medially.

***Male terminalia*** (Fig. 13): Epandrium with ca. 23 setae near posterior and ventral margins per side. Hypandrium lacking pubescence. Paramere with five sensilla medially. Aedeagus lacking pubescence.

***Measurements*** [holotype ♂ (range in 1♂ paratypes), in mm]: BL = 2.00 (2.07), ThL = 1.07 (1.17), WL = 2.06 (2.20), WW = 0.86 (0.90).

***Indices***: arb = 3/2 (3/2), avd = 1.14 (0.89), adf = 2.33 (3.00), flw = 2.33 (2.67), FW/HW = 0.45 (0.49), ch/o = 0.09 (0.08), prorb = 1.00 (1.11), rcorb = 0.38 (0.44), vb = 0.40 (0.29), dcl = 0.39 (0.35), presctl = 0.50 (0.55), sctl = 0.94 (damaged), sterno = damaged (damaged), orbito = 0.40 (0.50), dcp = 0.23 (0.33), sctlp = 0.890 (0.91), C = 3.33 (3.31), 4c = 0.71 (0.76), 4v = 1.59 (1.76), 5x = 1.60 (1.80), ac = 2.00 (2.17), M = 0.47 (0.53), C3F = 0.33 (0.46).

#### Etymology.

A combination of the Latin words *nigritus* and *limbus*, referring to the margins of tergites being nearly black.

#### Distribution.

China (Yunnan).

### Scaptodrosophila trivittata sp. n.

Taxon classificationAnimaliaDipteraDrosophilidae

http://zoobank.org/733F473B-B4D1-487C-A705-C5FCB51B9C2A

Figs 6B, D, F , 14

#### Holotype.

♂ (SCAU, No. 110984): CHINA: Mengdong, Cangyuan, Yunnan, alt. 1323 m, 6.v.2016, J Huang, YQ Liu.

#### Paratypes.

CHINA: 2♂ (SCAU, Nos 110985, 86), same data as holotype.

#### Diagnosis.

This species is similar to *S. fuscilimba* sp. n. in the pattern on the abdomen tergites, but can be distinguished from the latter by having the tergites V, VI yellowish brown, and lacking dark brown caudal bands (Fig. 6D, F); paramere triangle shaped in lateral view (Fig. 14C, D); gonopods small, concave medially in lateral view, bilobed apically (Fig. 14C, D); aedeagus rod-like, with pubescence, and broadened apically (Fig. 14C, D).

#### Description.

(♂) ***Head*** (Fig. 6B): Frons yellowish brown. Pedicel yellowish brown; first flagellomere yellowish, margins black. Facial carina yellowish, flat, 1/3 long as face.

***Thorax*** (Fig. 6D, F): Mesonotum yellowish brown. Acrostichal setulae in ca. ten regular rows. Prescutellar setae small. Scutellum yellowish brown. Pleura yellowish brown.

***Abdomen*** (Fig. 6D, F): Tergites II to IV yellow with dark brown caudal bands, the caudal bands narrowed dorsomedially; tergites V to VI yellow.

***Male terminalia*** (Fig. 14): Epandrium with ca. 16 setae near posterior and ventral margins per side. Surstylus with nine peg-like prensisetae. Hypandrium lacking pubescence. Paramere with ten sensilla medially.

***Measurements*** [holotype ♂ (range in 2♂paratypes), in mm]: BL = 2.00 (1.97–2.00), ThL = 0.83 (0.83), WL = 1.70 (1.70–1.93), WW = 0.67 (0.73–0.83).

***Indices***: arb = 4/2 (4/2), avd = 0.83 (0.83–1.00), adf = 2.00 (2.00), flw = 2.00 (2.00–2.33), FW/HW = 0.48 (0.50), ch/o = 0.13 (0.12–0.13), prorb = 0.86 (0.86), rcorb = 0.29 (0.29), vb = 0.30 (0.29–0.30), dcl = 0.46 (0.55), presctl = 0.38 (0.45), sctl = 1.17 (1.08), sterno = 0.50 (0.40), orbito = 0.38 (0.40–0.50), dcp = 0.33 (0.40–0.50), sctlp = 086 (1.00–1.17), C = 1.59 (1.59–1.88), 4c = 1.42 (1.23–1.42), 4v =2.17 (2.08–2.25), 5x = 3.00 (2.50), ac = 3.40 (2.67–3.40), M = 0.75 (0.77–0.83), C3F = 0.59 (0.59–0.69).

#### Etymology.

A combination of the Latin words *tri* and *vittatus*, referring to the three caudal bands on the tergites II to IV.

#### Distribution.

China (Yunnan).

### Scaptodrosophila ventriobscurata sp. n.

Taxon classificationAnimaliaDipteraDrosophilidae

http://zoobank.org/9703E5D0-A537-4672-8E6E-42326E16BCF5

Figs 7A, C, E , 15

#### Holotype.

♂ (SCAU, No. 111095): CHINA: Xincheng, Yingjiang, Yunnan, 24°46'N, 98°10'E, alt. 963 m, 18.viii.2016, HW Chen.

#### Paratypes.

CHINA: 2♂, 4♀ (SCAU, Nos 110996–111001), same data as holotype; 1♂ (SCAU, No. 111002), Wangtianshu, Mengla, Yunnan, 21°37'N, 101°36'E, alt. 760 m, 13.iv.2010, YR Su; 2♂ (SCAU, Nos 111003, 04), Menglun, Mengla, Yunnan, 680 m, 11.iv.2010, L Wu; 1♂ (SCAU, No. 111005), Husa, Longchuang, Yunnan, 24°27'N, 97°45'E, alt. 1227m, 21.viii.2016, L Gong; 1♂ (SCAU, No. 111006), Arboretum, Ruili, Yunnan, 24°1'N, 97°51'E, alt. 1174 m, 22.viii.2016, L Gong.

#### Diagnosis.

Surstylus with four large setae (Fig. 15A, B); cercus with six long peg-like prensisetae (Fig. 15A, B); gonopods large, with an acute protection apically (Fig. 15C, D); aedeagus rod-like, elliptically broadened apically (Fig. 15C, D).

#### Description.

(♂, ♀) ***Head*** (Fig. 7A): Frons yellowish brown. Pedicel yellowish brown; first flagellomere yellowish, margins black. Facial carina yellowish, 1/3 long as face.

***Thorax*** (Fig. 7C, E): Mesonotum yellowish brown. Acrostichal setulae in ca. eight to ten regular rows. Prescutellar setae small. Scutellum yellowish brown. Pleura yellowish, with a brown longitudinal stripe.

***Abdomen*** (Fig. 7C, E): Tergites II to V yellowish with broad black caudal bands, the caudal bands on tergites II and III narrowed medially; tergites VI black.

***Male terminalia*** (Fig. 15A–D): Epandrium with ca. 11 setae near posterior and ventral margins per side. Surstylus with 11 peg-like prensisetae, and pubescence anterior. Hypandrium with pubescence. Paramere large, with seven sensilla subbasally.

***Female terminalia*** (Fig. 15E): Oviscapt very broadened apically, with a single apical trichoid ovisensillum, and 25 ovisensilla.

***Measurements*** [holotype ♂ (range in 4♂, 4♀ paratypes), in mm]: BL = 2.33 (1.90–2.23, 2.33–2.50), ThL = 1.00 (0.83–1.33, 0.90–1.03), WL = 2.00 (1.63–1.87, 1.90–2.07), WW = 0.93 (0.73–0.97, 0.87–0.97).

***Indices***: arb = 4/2 (3–4/2), avd = 1.00 (0.83–0.86), adf = 2.00 (1.67–2.33), flw = 1.67 (1.50–2.00), FW/HW = 0.44 (0.39–0.49), ch/o = 0.10 (0.10–0.20), prorb = 0.80 (0.63–1.00), rcorb = 0.50 (0.50–0.71), vb = 0.43 (0.38–0.50), dcl = 0.69 (0.59–0.83), presctl = 0.31 (0.22–0.42), sctl = 0.94 (0.94–1.06), sterno = 0.80 (0.67–0.87), orbito = 0.60 (0.50–0.63), dcp = 0.43 (0.40–0.55), sctlp = 0.75 (0.75–1.00), C = 1.63 (1.50–2.00), 4c = 1.46 (1.14–1.70), 4v =2.31 (2.21–2.60), 5x = 2.50 (2.25–3.00), ac = 3.17 (2.67–3.00), M = 0.77 (0.79–0.91), C3F = 0.68 (0.63–0.73).

#### Etymology.

A combination of the Latin words *ventris* and *obscuratus*, referring to the black tergites.

#### Distribution.

China (Yunnan).

### Scaptodrosophila zebrina sp. n.

Taxon classificationAnimaliaDipteraDrosophilidae

http://zoobank.org/32789355-9D24-444B-991E-6DA8387F43E0

Figs 7B, D, F , 16

#### Holotype.

♂ (SCAU, No. 110007) CHINA: Mengyuan, Mengla, Yunnan, 21°47'N, 101°22'E, alt. 995 m, 7.viii.2016, HW Chen.

#### Paratypes.

CHINA: 3♂, 4♀ (SCAU, Nos 110008–14), same data as holotype, HW Chen, L Gong, YQ Liu; 1♂, 1♀ (SCAU, Nos 110015, 16), Wangtianshu, Mengla, Yunnan, alt. 580 m, 22.iv.2007, HW Chen; 2♀ (SCAU, Nos 110017, 18), Wangtianshu, Mengla, Yunnan, alt. 760 m, 27.viii.2012, HW Chen.

#### Diagnosis.

Frons yellowish brown with two brown longitudinal stripes (Fig. 7B); mesonotum yellowish brown with six dark brown longitudinal stripes (Fig. 7D, F); gonopods long in lateral view (Fig. 16D); aedeagus rectangle shaped in lateral view (Fig. 16D).

#### Description.

(♂, ♀) ***Head*** (Fig. 7B): Pedicel yellowish brown; first flagellomere yellowish, margins black. Facial carina yellowish, broad, 1/3 long as face.

***Thorax*** (Fig. 7D, F): Mesonotum yellowish brown. Acrostichal setulae in ca. ten regular rows. Prescutellar setae small. Scutellum yellowish brown. Pleura yellowish.

***Abdomen*** (Fig. 7D, F): Tergites II to VI yellow with black caudal bands, the caudal bands on tergites interrupted medially.

***Male terminalia*** (Fig. 16A–D): Epandrium with ca. 12 setae near posterior and ventral margins per side. Surstylus with seven peg-like prensisetae. Hypandrium lacking pubescence. Paramere long, with two sensilla distally.

***Female terminalia*** (Fig. 16E): Oviscapt with five subapical trichoid ovisensilla, and 13 ovisensilla.

***Measurements*** [holotype ♂ (range in 4♂, 5♀ paratypes), in mm]: BL = 2.17 (2.20–2.50, 2.33–2.67), ThL = 1.13 (1.10–1.17, 0.90–1.53), WL = 2.13 (2.10–2.20, 2.20–2.47), WW = 0.90 (0.90–1.20, 0.90–1.00).

***Indices***: arb = 4/2 (3–4/2), avd = 1.00 (0.75–1.00), adf = 1.75 (1.75–2.67), flw = 1.50 (1.50–2.00), FW/HW = 0.43 (0.40–0.47), ch/o = 0.09 (0.09–0.10), prorb = 0.80 (0.64–0.90), rcorb = 0.40 (0.27–0.45), vb = 0.29 (0.25–0.43), dcl = 0.57 (0.55–0.71), presctl = 0.38 (0.32–0.62), sctl = 1.00 (0.95–1.18), sterno = 0.50 (0.41–0.64), orbito = 0.60 (0.33–0.50), dcp = 0.56 (0.47–1.00), sctlp = 0.89 (0.67–1.00), C = 2.50 (2.06–2.65), 4c = 0.94 (0.94–1.13), 4v = 1.82 (1.81–2.00), 5x = 1.50 (1.29–1.80), ac = 3.20 (3.00–3.60), M = 0.53 (0.47–0.63), C3F = 0.63 (0.56–0.67).

#### Etymology.

From the Latin word *zebrinus*, referring to the brown longitudinal stripes on the mesonotum.

#### Distribution.

China (Yunnan).

##### Key to species of the *coracina* group

**Table:** 

1	Body yellowish brown to black; arista with three or four dorsal and two ventral branches in addition to terminal bifurcation; facial carina narrow and flat; prescutellar setae usually large, as long as anterior dorsocentral setae; hypandrium usually with a pair of very large paramedian setae	***coracina* group**...**2**
2	Frons yellowish brown with two brown longitudinal stripes (Fig. 7B); mesonotum yellowish brown with six dark brown longitudinal stripes (Fig. 7D, F)	***S. zebrina* sp. n.**
–	Frons lacking longitudinal stripes; mesonotum lacking longitudinal stripes	**3**
3	Hypandrium with pubescence	**4**
–	Hypandrium lacking pubescence	**6**
4	Body brown or black (Fig. 3C–F); paramere with pubescence completely (Fig. 8C, D); aedeagus short, rod-like, with one fan-like membrane apically (Fig. 8C, D)	***S. coracina***
–	Body yellowish brown; paramere sometimes with pubescence; aedeagus lacking membrane apically	**5**
5	Pleura yellowish, with one brown longitudinal stripe (Fig. 7E); surstylus with four large setae (Fig. 15A, B); cercus with six long peg-like prensisetae (Fig. 15A, B); paramere elliptical shaped in lateral view, lacking pubescence apically (Fig. 15C, D); aedeagus rod-like, elliptically broadened apically (Fig. 15C, D)	***S. ventriobscurata* sp. n.**
–	Pleura yellowish brown, lacking longitudinal stripe (Fig. 4F); surstylus lacking large setae (Fig. 10A, B); cercus lacking long peg-like prensisetae (Fig. 10A, B); paramere dolabriform in lateral view, with pubescence apically (Fig. 10C, D); aedeagus very broad and finely curved apically in lateral view (Fig. 10D)	***S. fusciventricula* sp. n.**
6	Gonopods bilobed apically	**7**
–	Gonopods fused completely apically	**8**
7	Pleura yellowish brown, lacking longitudinal stripe (Fig. 6F); abdomen tergites yellow, tergites II to IV yellow with dark brown caudal bands (Fig. 6D, F); aedeagus rod-like, with pubescence and broadened apically (Fig. 14C, D)	***S. trivittata* sp. n.**
–	Pleura yellowish brown, with two brown longitudinal stripes (Fig. 5F); abdomen tergites yellowish brown, tergites II to V yellow with dark brown caudal bands, tergite VI dark brown (Fig. 5D, F); aedeagus quadrangle in lateral view, lacking pubescence (Fig. 12D)	***S. longispinata* sp. n.**
8	Paramere with one small projection subbasally (Fig. 13D); gonopods with one small projection distally (Fig. 13D); aedeagus triangle shaped in lateral view (Fig. 13D)	***S. nigrolimbata* sp. n.**
–	Paramere lacking projection subbasally; gonopods lacking projection distally; aedeagus inverted triangle shaped in lateral view	**9**
9	Tergite VI yellowish brown, with dark brown caudal bands (Fig. 5C, E); paramere small, finger like in lateral view (Fig. 11D); aedeagus round dorsally in lateral view (Fig. 11D)	***S. helvpecta* sp. n.**
–	Tergite VI yellow, lacking band (Fig. 4C); paramere curved strongly in lateral view (Fig. 9D); aedeagus tapering dorsally in lateral view (Fig. 9D)	***S. fuscilimba* sp. n.**

## Supplementary Material

XML Treatment for Scaptodrosophila coracina species group

XML Treatment for Scaptodrosophila coracina

XML Treatment for Scaptodrosophila fuscilimba

XML Treatment for Scaptodrosophila fusciventricula

XML Treatment for Scaptodrosophila helvpecta

XML Treatment for Scaptodrosophila longispinata

XML Treatment for Scaptodrosophila nigrolimbata

XML Treatment for Scaptodrosophila trivittata

XML Treatment for Scaptodrosophila ventriobscurata

XML Treatment for Scaptodrosophila zebrina
